# 25-Hydroxyvitamin D and Its Relationship with Autonomic Dysfunction Using Time- and Frequency-Domain Parameters of Heart Rate Variability in Korean Populations: A Cross-Sectional Study

**DOI:** 10.3390/nu6104373

**Published:** 2014-10-16

**Authors:** Young Jin Tak, Jeong Gyu Lee, Yun Jin Kim, Sang Yeoup Lee, Byung Mann Cho

**Affiliations:** 1 Department of Family Medicine, Pusan National University School of Medicine, 602-739 Busan, Korea; E-Mails: 03141998@hanmail.net (Y.J.T.); yujkim@pusan.ac.kr (Y.J.K.); 2 Biomedical Research Institute, Pusan National University Hospital, 602-739 Busan, Korea; 3 Medical Education Unit and Medical Research Institute, Pusan National University School of Medicine, 626-870 Yangsan, Korea; E-Mail: saylee@pnu.edu; 4 Family Medicine Clinic and Research Institute of Convergence of Biomedical Science and Technology, Pusan National Yangsan Hospital, 626-789 Yangsan, Korea; 5 Department of Preventive Medicine and Occupational Medicine, Pusan National University School of Medicine, 626-770 Yangsan, Korea; E-Mail: bmcho@hanmail.net

**Keywords:** heart rate variability, vitamin D, 25-hydroxyvitamin D

## Abstract

Previous studies have demonstrated that reduced heart rate variability (HRV) and hypovitaminosis D are associated with cardiovascular disease (CVD). However, few reports have investigated the effects of vitamin D on HRV. This cross-sectional study analyzed serum 25-hydroxyvitamin D (25(OH)D) and HRV indices using 5-min R-R interval recordings with an automatic three-channel electrocardiography in healthy subjects (103 males and 73 females). Standard deviation of N-N interval (SDNN), square root of mean squared differences of successive N-N intervals (RMSSD), total power (TP), very low frequency (VLF), low frequency (LF), and high frequency (HF) were reported. The mean age of subjects was 55.3 ± 11.3 years and the mean 25(OH)D level was 21.2 ± 9.9 ng/mL. In a multiple linear regression model, 25(OH)D was positively correlated with SDNN (β = 0.240, *p* < 0.002), and LF (β = 0.144, *p* = 0.044). Vitamin D deficiency (25(OH)D < 15 ng/mL) was associated with decreased SDNN (<30 m/s) (OR, 3.07; 95% confidence interval (CI), 1.32–7.14; *p* = 0.014) after adjusting for covariates. We found that lower 25(OH)D levels were associated with lower HRV, suggesting a possible explanation for the higher risk of CVD in populations with hypovitaminosis D.

## 1. Introduction

In addition to its role in bone and calcium metabolism, vitamin D has important prohormone functions in a wide range of clinical processes, including antiproliferative, prodifferentiative and immunomodulatory actions [1]. Vitamin D deficiency has become the most widespread nutritional disorder in the modern world because of decreased sunlight exposure, increasing obesity, and changes in dietary habits [2]. Numerous studies have demonstrated that hypovitaminosis D is related to increased risks of cardiovascular diseases (CVD), metabolic dysfunctions, and high all-cause mortality in the general population [3]. Specifically, vitamin D metabolites and its metabolism gene CYP24A1 are associated with coronary atherosclerosis and calcification [4]. These observations support the hypothesis that low vitamin D levels influence the activity of the cardiovascular system and result in a dysfunctional cardiac autonomic nervous system (ANS). Chan *et al.* reported that patients with chronic kidney disease (impaired vitamin D synthesis) showed poor cardiosympathovagal activity characterized by a withdrawal of inhibitory vagal activity [5]. However, few studies exploring the association between vitamin D and cardiac autonomic function in healthy people have been reported.

Heart rate interval changes are the result of the ANS dynamically regulating the body’s response to internal and external stimuli. The balance of the ANS activity reflects physiological, hormonal and psychological stability [6]. Heart rate variability (HRV) analysis is based on the measurement of interval variability between R waves (RR intervals) and qualitative and quantitative assessments that represent the balance of the cardiovascular system via ANS control [7]. As an established tool in cardiology studies, HRV is used currently for a wide range of clinical conditions from psychiatric illnesses to internal organ pathologies. Increased HRV reflects a healthy ANS able to react appropriately to changing environmental circumstances [8], whereas decreased HRV is a sign of autonomic inflexibility and heart disease that may precede systemic problems (e.g., inflammatory-mediated atherosclerosis and ventricular fibrillation) [9]. Recent research has shown that a decreased HRV is associated with risk factors for CVD, heart failure, and sudden cardiac death (SCD) [10].

To understand the effects of vitamin D deficiency on the heart, the association between vitamin D deficiency and HRV indices must be examined in terms of clinical importance. To date, few studies have explored the effects of vitamin D on HRV in healthy individuals. Therefore, in the present study we examined the relationship between serum vitamin D levels and HRV and hypothesized that lower serum vitamin D levels are associated with lower HRV parameters.

## 2. Experimental Section

### 2.1. Study Population

We conducted a cross-sectional study based on data extracted from our hospital medical records. Data on healthy subjects over 20 years of age who underwent a comprehensive medical examination including HRV and serum vitamin D levels from July 2012 through February 2014 (*n* = 176) were collected. We selected participants who underwent both HRV and blood test including serum vitamin D level on the same day. Criteria for exclusion were as follows: missing data about vitamin D level and HRV; chronic diseases that can influence the ANS, including diabetes mellitus (DM), hypertension (HTN), arrhythmia, heart failure, coronary heart disease, depression, and panic disorder; receiving medications such as angiotensin-converting enzyme inhibitors, β-receptor agonists or antagonists, calcium channel blockers, or anticholinergics, which can influence the ANS; mean heart rate of more than 100 or less than 50 beats per minute; presence of other health conditions that can affect the vitamin D level, such as cancer, parathyroid gland disease, liver disease, epilepsy, inflammatory bowel disease, malabsorption, celiac disease, gastric bypass, bowel surgery; and regular administration of vitamin D supplements within the previous 3 months. The study protocol was approved by the Institutional Review Board of Pusan National University Hospital (IRB No. E-2014064).

### 2.2. Data Collection

Subjects were interviewed by a physician regarding their medical history, smoking status, alcohol consumption and exercise habits. The subjects were divided into nonsmokers or current smokers. The frequency of drinking per week, beverage type, and amount consumed were recorded. An alcohol drinker was defined as a subject consuming >20 g of alcohol per day [11]. Regular exercise was defined as subjects exercising more than once per week at moderate or greater intensity [12]. A trained examiner measured the height and body weight of the patients wearing a light gown without shoes to the nearest 0.1 cm and 0.1 kg, respectively, using an HM-300 (Fanics Co. Ltd., Busan, Korea). Body mass index (BMI) was calculated by dividing the weight in kg by the height in meters squared (kg/m^2^). Waist circumference (WC) was measured at the smallest distance between the lower margin of the rib cage and the iliac crest, at the end of normal expiration and to the nearest 0.1 cm. Blood pressure (BP) was assessed twice while the subjects were seated using an automated BP measurement device (BP-203RV II, Colin Corp., Aichi, Japan) with a 5 min rest in between and the two results were averaged. The blood sample was drawn from the antecubital vein between 8 and 9 AM after a 12-h overnight fast, and was subsequently analyzed at a certified laboratory using an automatic blood analyzer (Hitachi 7600-110 chemical analyzer, Hitachi Co. Ltd., Tokyo, Japan). Fasting plasma glucose (FPG) was evaluated using the glucose oxidase method with a Synchron LX 20 (Beckman Coulter, Fullerton, CA, USA). Total cholesterol (TC) was calculated using an autoanalyzer with the enzymatic colorimetric method Toshiba TBA200FR (Toshiba Co. Ltd., Tokyo, Japan). Serum creatinine (sCr) was analyzed by kinetic colorimetric assay based on a modified Jaffe method using a commercial enzymatic kit (Modular-DP, Roche, Basel, Switzerland). Glomerular filtration rate estimated (eGFR) from sCr was reported for determination of kidney function because serum 25(OH)D level is dependent on kidney function. eGFR was inspected using the following Equation (1) from the Modification of Diet in Kidney Dysfunction Study (MDRD) [13].


[eGFR (mL/min/1.73 m^2^) = 186.3 × sCr^−1.154^ × age^−0.203^ (× 0.742 for women)] (1)

The seasonality of the blood test was categorized into four groups: spring (March–May), summer (June–August), fall (September–November) and winter (December–February).

### 2.3. Measurement of Serum 25-Hydroxyvitamin D

Vitamin D status is commonly assessed by the serum 25-hydroxyvitamin D (25(OH)D) level because it can reflect vitamin D derived from both dietary intake and dermal production [14]. To examine the serum 25(OH)D level, blood samples were drawn from the antecubital vein after a 12-h overnight fast during a routine health examination. The serum 25(OH)D level was assessed as total 25(OH)D (vitamin D2 + vitamin D3) with a chemiluminescence immunoassay using the LIAISON^®^25 OH Vitamin D TOTAL Assay (DiaSorin Inc., Stillwater, MN, USA) at the Eone Reference Laboratory (Seoul, Korea), which guaranteed intra-assay and inter-assay coefficients of variation less than 10%. According to recent clinical guidelines, vitamin D deficiency was defined as a serum 25(OH)D level < 15 ng/mL. This threshold is based on a study showing serum 25(OH)D < 15 ng/mL is correlated with an increased risk of incident CVD [15].

### 2.4. Heart Rate Variability Measurements

To measure the HRV parameters, the subjects did not consume caffeine (*i.e.*, tea or coffee) and rested for 30 min before the study. Subsequently a three-channel (both wrists and left ankle) electrocardiographic recording was conducted for 5 min with the subjects sitting in a quiet room and was automatically computed using SA-6000P (Medicore Inc., Seoul, Korea). This device meets the assessment standards and physiological interpretation as well as bio-signal processing algorithms created by the Task Force of the European Society of Cardiology and the North American Society of Pacing and Electrophysiology [16]. During the test, subjects were instructed to breathe naturally without any conscious respiratory manipulation for a more accurate analysis. As the time domain index, mean heart rate (MHR), standard deviation of the N-N interval (SDNN) and the square root of the mean squared differences of successive N-N intervals (RMSSD) were examined. SDNN reflects the overall cyclic components of HRV during the recording period and RMSSD reflects an estimated parasympathetic regulation of the heart [16,17]. As the frequency domain index, total power (TP; total power for 5 min including VLF, LF and HF), very low frequency (VLF; frequency strength of 0.04–0.15 Hz), low frequency (LF; frequency strength of 0–0.04 Hz), high frequency (HF; frequency strength of 0.15–0.4 Hz) and the low-frequency/high-frequency ratio (LF/HF ratio) were reported. TP reflects mainly the level of the autonomic nervous activities and VLF band is an additional indicator of sympathetic function. The LF component reflects the complex interaction between sympathetic and parasympathetic activities of heart rate and baroreceptor activity. The HF parameter assesses parasympathetic activity and the LF/HF ratio is an overall relative balance estimate of the autonomic activity [16,17].

### 2.5. Statistical analysis

SPSS version 18.0 (SPSS Inc., Chicago, IL, USA) was used for statistical analyses. Unless stated otherwise, continuous variables were expressed as means ± standard deviations. Categorical variables were presented as frequencies and proportions. Because TP, HF, LF and LF/HF ratio were right-skewed, they were log-transformed to gain a normal distribution. The subjects were divided into deficiency and non-deficiency groups based on serum 25(OH)D levels. To compare variables between the 25(OH)D non-deficiency group and 25(OH)D deficiency group, the chi-square test was computed to investigate categorical variables and the independent *t*-test for continuous variables. Seasonal variations in 25(OH)D, SDNN, and RSMMD values were assessed using analysis of variance (ANOVA). In addition, the Mann-Whitney *U-*test was used for comparison of SDNN and RSMMD values according to 25(OH)D status in each season. A linear regression analysis was conducted to assess relations between HRV parameters and 25(OH)D levels. In regression test, *p*-values were corrected by the Bonferroni correction for multiple comparisons. Therefore, a *p*-value ˂ 0.00625 (0.05/8) was considered to indicate statistical significance. Age, sex, and seasons of 25(OH)D measurement were included as covariate factors in a multivariate analysis. In addition, an SDNN cutoff value separating better and worse outcomes in the order of 30 m/s was used [18]. The odds ratio (OR) of SDNN < 30 m/s was calculated using a multiple logistic regression model among the 25(OH)D status groups after adjusting for confounders.

## 3. Results

### 3.1. Patients’ Characteristics

**Figure 1:**
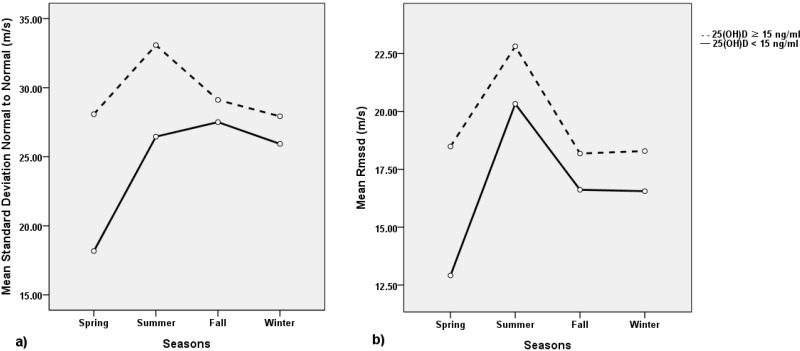
Seasonal variation in mean levels of SDNN (m/s) (**a**) and RMSSD (m/s) (**b**) according to 25-hydroxyvitamin D status. Abbreviation: SDNN = standard deviation of normal to normal, RMSSD = square root of the mean of sum of the square of differences between adjacent N-N intervals, 25(OH)D = 25-hydroxyvitamin D.

A total of 176 subjects (103 males and 73 females) 20–80 years of age (average age 55.3 ± 11.3 years) participated in this study. The mean 25(OH)D value was 21.2 ± 9.9 ng/mL. Twenty-eight percent of all subjects were deficient in vitamin D (25(OH)D < 15 ng/mL); only 14.7% of subjects were vitamin D sufficient (25(OH)D ≥ 30 ng/mL). Table 1 shows the subjects’ clinical characteristics according to the 25(OH)D status. The 25(OH)D deficient group had a higher proportion of females (62% *vs.* 33.3%, *p* = 0.001) and lower SDNN value (25.3 ± 8.4 m/s *vs.* 30.2 ± 16.2 m/s, *p* = 0.044) than the non-deficient 25(OH)D group. The RMSSD, TP, VLF, LF, and LF/HF ratio were also slightly lower in the 25(OH)D deficient group compared to the 25(OH)D non-deficient group. However, these differences were not statistically significant (*p* > 0.05). When evaluating seasonal variation, the 25(OH)D level was highest in the fall and lowest in the spring (23.26 ± 11.9 *vs.* 18.55 ± 6.7 ng/mL). Similarly, SDNN and RMSSD values were the lowest in the spring, although not statistically significant (Figure 1). No evidence of significant seasonal variation in the SDNN and RMSSD values according to 25(OH)D status was detected.

**Table 1:** Characteristics of study subjects according to 25-hydroxyvitamin D status.

Variables	Total (*n* = 176)	25-Hydroxyvitamin D Status		*p*-Value *
		Non-Deficient Group 25(OH)D ≥ 15 ng/mL (*n* = 126)	Deficient Group 25(OH)D < 15 ng/mL (*n* = 50)	
Female (%)	73 (41.5)	42 (33.3)	31 (62.0)	0.001
Age (year)	55.3 ± 11.3	54.9 ± 11.7	55.9 ± 10.9	0.592
BMI (kg/m^2^)	24.0 ± 3.4	24.1 ± 3.2	23.8 ± 3.8	0.566
WC (cm)	83.8 ± 9.6	84.2 ± 9.4	82.2 ± 10.3	0.561
SBP (mmHg)	115.9 ± 12.7	115.2 ± 12.4	116.9 ± 13.3	0.510
DBP (mmHg)	71.4 ± 8.2	71.5 ± 7.7	71.5 ± 9.0	0.982
TC (mg/dL)	198.1 ± 36.7	199.8 ± 37.5	193.7 ± 34.5	0.323
FPG (mg/dL)	85.9 ± 11.9	86.1 ± 12.3	85.4 ± 11.3	0.753
eGFR (mL/min/1.73 m^2^)	90.1 ± 19.5	88.5 ± 1.8	94.1 ± 2.4	0.062
Current smoker (%)	49 (27.8)	37 (29.4)	12 (24.0)	0.777
Alcohol drinker (%)	43 (24.4)	34 (27.0)	9 (18.0)	0.247
Regular exerciser (%)	75 (42.6)	54 (42.9)	21 (42.0)	0.527
25(OH)D (ng/mL)	21.2 ± 9.9	25.2 ± 9.0	11.4 ± 2.5	<0.001
HRV time domain index				
Mean heart rate (bpm)	70.0 ± 10.0	69.7 ± 10.7	70.6 ± 8.6	0.302
SDNN (m/s)	28.9 ± 14.5	30.2 ± 16.2	25.3 ± 8.4	0.044
RMSSD (m/s)	19.3 ± 13.2	20.0 ± 14.6	17.3 ± 8.3	0.235
HRV frequency domain index				
Total power (log m/s^2^)	6.13 ± 0.9	6.2 ± 0.9	6.0 ± 0.8	0.253
VLF (log m/s^2^)	5.43 ± 1.0	5.5 ± 1.0	5.3 ± 1.0	0.272
LF (log m/s^2^)	4.64 ± 1.2	4.7 ± 1.2	4.4 ± 1.1	0.120
HF (log m/s^2^)	4.01 ± 1.2	3.9 ± 1.2	4.0 ± 1.1	0.818
LF/HF ratio	4.18 ± 1.5	4.8 ± 1.2	2.6 ± 3.7	0.212

Abbreviation: BMI = body mass index, DBP = diastolic blood pressure, FPG = fasting plasma glucose, HF = high frequency, HRV = heart rate variability, LF = low frequency, LF/HF ratio = low frequency/high frequency ratio, RMSSD = Square root of the mean of sum of the square of differences between adjacent N-N interval, SBP = systolic blood pressure, SDNN = standard deviation of normal to normal, TC = total cholesterol, VLF = very low frequency, WC = waist circumference, 25(OH)D = 25-hydroxyvitamin D; Values are expressed as frequencies (%) or means ± standard deviation (SD) unless otherwise indicated; * Calculated by chi-square test or *t*-test.

### 3.2. Associations of 25(OH)D Levels and HRV Time Domain

Table 2 presents a linear regression analysis between 25(OH)D levels and HRV time domain indices. MHR showed significantly negative linear relations with age (β = −0.419, *p* < 0.001), but was not associated with 25(OH)D levels (*p* = 0.322). SDNN was positively associated with 25(OH)D levels (β = 0.272, *p* < 0.001). In the multiple linear regression analysis, association between 25(OH)D and SDNN was sustained after adjusting for age, sex, and season of 25(OH)D measurement (β = 0.240, *p* = 0.002). However, 25(OH)D levels did not show any statistical relation with RMSSD (*p* > 0.00625).

### 3.3. Associations of 25(OH)D Levels and HRV Frequency Domain

Table 3 shows a linear regression analysis between 25(OH)D levels and HRV frequency domain indices. In a univariate analysis, 25(OH)D levels showed a significantly positive relation with LF (β = 0.234, *p* = 0.002). However this association became non-significant after adjusting for covariates (*p* > 0.00625). In contrast, 25(OH)D levels did not show a significant association with TP, HF and LF/HF ratio. After adjusting for age, gender, seasons, WC, BMI, alcohol consumption, smoking status, regular exercise, BP, TC, FPG, and eGFR, vitamin D deficiency (25(OH)D < 15 ng/mL) was independently associated with low SDNN (<30 m/s), with an OR of 3.07 (95% confidence interval (CI), 1.32–7.14). However, vitamin D deficiency was not associated with low RMSSD (<10 m/s), with an OR 1.86 (95% CI, 0.70–4.96) (Table 4).

**Table 2:** Associations of 25-hydroxyvitamin D with heart rate variability time domain indices using univariate and multivariate linear regression analyses.

	Mean Heart Rate (bpm)			SDNN (m/s)			RMSSD (m/s)		
	B ± SE	β	*p*-Value	B ± SE	β	*p*-Value	B ± SE	β	*p*-Value
*Univariate analysis*									
Male	−1.014 ± 1.558	−0.049	0.516	−1.122 ± 2.232	−0.038	0.616	−1.890 ± 2.024	−0.071	0.352
Age (year)	−0.370 ± 0.061	−0.419	<0.001	−0.158 ± 0.095	−0.125	0.099	0.036 ± 0.087	0.032	0.676
Winter-Spring	1.325 ± 1.625	0.062	0.416	−3.745 ± 2.314	−0.121	0.107	−2.899 ± 2.112	−0.103	0.172
BMI (kg/m^2^)	−0.503 ± 0.226	−0.167	0.027	0.204 ± 0.327	0.047	0.533	0.358 ± 0.297	0.091	0.228
WC (cm)	−0.124 ± 0.079	−0.118	0.119	0.056 ± 0.114	0.037	0.623	0.137 ± 0.103	0.100	0.187
SBP (mmHg)	0.056 ± 0.061	0.070	0.358	−0.009 ± 0.087	−0.008	0.917	0.025 ± 0.079	0.024	0.751
DBP (mmHg)	0.144 ± 0.094	0.115	0.129	0.025 ± 0.136	0.014	0.857	0.063 ± 0.124	0.038	0.613
TC (mg/dL)	−0.012 ± 0.021	−0.045	0.561	−0.008 ± 0.030	−0.021	0.784	−0.020 ± 0.028	−0.056	0.464
FPG (mg/dL)	−0.081 ± 0.063	−0.098	0.201	−0.052 ± 0.093	−0.043	0.576	−0.012 ± 0.085	−0.011	0.886
eGFR (mL/min/1.73 m^2^)	0.027 ± 0.039	0.053	0.486	−0.066 ± 0.056	−0.089	0.241	−0.029 ± 0.051	−0.043	0.571
Current smoker	−1.588 ± 1.704	−0.070	0.353	−0.140 ± 2.445	−0.004	0.954	1.645 ± 2.244	0.056	0.460
Alcohol drinker	−1.205 ± 1.779	−0.051	0.499	−1.292 ± 2.549	−0.038	0.613	−0.785 ± 2.323	−0.026	0.736
Regular exerciser	−3.238 ± 1.524	−0.159	0.035	4.185 ± 2.187	0.143	0.057	5.223 ± 1.974	0.196	0.009
25(OH)D (ng/mL)	0.077 ± 0.077	0.075	0.322	0.396 ± 0.107	0.272	<0.001	0.220 ± 0.099	0.165	0.028
*Multivariate analysis*									
Male	−0.549 ± 1.429	−0.027	0.701	−1.012 ± 2.159	−0.034	0.640	−1.997 ± 2.008	−0.075	0.321
Age (year)	−0.369 ± 0.063	−0.418	< 0.001	−0.095 ± 0.095	−0.075	0.317	0.078 ± 0.088	0.068	0.380
Winter-Spring	0.856 ± 1.526	0.040	0.575	−2.576 ± 2.305	−0.083	0.265	−1.981 ± 2.144	−0.071	0.357
25(OH)D (ng/mL)	−0.007 ± 0.074	−0.007	0.920	0.350 ± 0.111	0.240	0.002	0.220 ± 0.104	0.166	0.035
*R*^2^	0.178			0.086			0.042		

Abbreviation: B = unstandardized regression coefficients, β = standardized regression coefficients, DBP = diastolic blood pressure, eGFR = estimated glomerular filtration rate, FPG = fasting plasma glucose, RMSSD = Square root of the mean of sum of the square of differences between adjacent N-N interval, SBP = systolic blood pressure, SDNN = standard deviation of normal to normal, SE = standard error, TC = total cholesterol, WC = waist circumference, 25(OH)D = 25-hydroxyvitamin D; Age, sex, and seasons of test were included as covariate factors in a multivariate analysis; A *p*-value ˂ 0.00625 (0.05/8) was considered to indicate statistical significance because *p*-value was corrected by the Bonferroni correction for multiple comparisons.

**Table 3:** Associations of 25-hydroxyvitamin D with heart rate variability frequency domain indices using univariate and multivariate linear regression analyses.

	Total Power (log m/s^2^)			HF (log m/s^2^)			LF (log m/s^2^)			VLF (log m/s^2^)			LF/HF Ratio (log)		
	B ± SE	β	*p*-Value	B ± SE	β	*p*-Value	B ± SE	β	*p*-Value	B ± SE	β	*p*-Value	B ± SE	β	*p*-Value
Univariate analysis															
Male	−0.090 ± 0.136	−0.050	0.507	−0.272 ± 0.187	−0.110	0.147	0.124 ± 0.180	0.052	0.491	−0.106 ± 0.157	−0.051	0.500	0.392 ± 0.176	0.167	0.027
Age (year)	−0.027 ± 0.005	−0.349	<0.001	−0.015 ± 0.008	−0.139	0.067	−0.043 ± 0.007	−0.421	<0.001	−0.024 ± 0.006	−0.274	<0.001	−0.028 ± 0.007	−0.280	<0.001
Winter-Spring	0.004 ± 0.142	0.002	0.979	−0.125 ± 0.197	−0.048	0.526	−0.123 ± 0.188	−0.049	0.515	0.146 ± 0.166	0.067	0.379	1.852 ± 1.690	0.083	0.275
BMI (kg/m^2^)	−0.001 ± 0.020	−0.004	0.955	0.011 ± 0.028	0.031	0.680	−0.006 ± 0.026	−0.017	0.823	−0.011 ± 0.023	−0.037	0.630	−0.018 ± 0.026	−0.051	0.497
WC (cm)	−0.004 ± 0.007	−0.046	0.546	−0.001 ± 0.010	−0.004	0.958	−0.007 ± 0.009	−0.057	0.450	−0.006 ± 0.008	−0.053	0.482	−0.007 ± 0.009	−0.057	0.455
SBP (mmHg)	−0.004 ± 0.005	−0.060	0.425	0.001 ± 0.007	−0.002	0.974	−0.008 ± 0.007	−0.083	0.272	−0.005 ± 0.006	−0.056	0.462	−0.008 ± 0.007	−0.083	0.275
DBP (mmHg)	0.001 ± 0.008	0.011	0.886	0.001 ± 0.011	−0.002	0.975	−0.007 ± 0.011	−0.045	0.550	0.003 ± 0.010	0.022	0.774	−0.006 ± 0.011	−0.044	0.564
TC (mg/dL)	−0.001 ± 0.002	−0.014	0.853	−0.002 ± 0.003	−0.056	0.462	0.001 ± 0.002	−0.009	0.909	0.001 ± 0.002	0.009	0.907	0.002 ± 0.002	0.054	0.484
FPG (mg/dL)	−0.007 ± 0.006	−0.100	0.189	−0.012 ± 0.008	−0.121	0.113	−0.014 ± 0.007	−0.146	0.055	−0.004 ± 0.007	−0.048	0.527	−0.002 ± 0.007	−0.019	0.804
eGFR (mL/min/1.73 m^2^)	0.001 ± 0.003	0.026	0.728	0.008 ± 0.005	0.124	0.100	0.004 ± 0.005	0.069	0.361	−0.001 ± 0.004	−0.028	0.716	−0.004 ± 0.004	−0.061	0.420
Current smoker	−0.070 ± 0.149	−0.036	0.639	−0.196 ± 0.206	−0.072	0.353	0.021 ± 0.197	0.008	0.917	−0.065 ± 0.174	−0.028	0.711	0.223 ± 0.195	0.086	0.256
Alcohol drinker	−0.043 ± 0.156	−0.021	0.783	−0.197 ± 0.215	−0.069	0.360	−0.024 ± 0.206	−0.009	0.906	−0.016 ± 0.182	−0.007	0.929	0.173 ± 0.204	0.064	0.399
Regular exerciser	0.105 ± 0.135	0.059	0.437	0.243 ± 0.186	0.098	0.194	0.137 ± 0.178	0.058	0.441	−0.025 ± 0.158	−0.012	0.874	−0.104 ± 0.177	−0.044	0.559
25(OH)D (ng/mL)	0.017 ± 0.007	0.195	0.010	0.006 ± 0.009	0.050	0.510	0.028 ± 0.009	0.234	0.002	0.019 ± 0.008	0.186	0.013	0.021 ± 0.009	0.179	0.017
Multivariate analysis															
Male	−0.057 ± 0.128	−0.032	0.656	−0.261 ± 0.187	−0.105	0.164	0.172 ± 0.162	0.073	0.290	−0.069 ± 0.151	−0.033	0.647	0.430 ± 0.168	0.183	0.012
Age (year)	−0.025 ± 0.006	−0.320	<0.001	−0.014 ± 0.008	−0.133	0.087	−0.040 ± 0.007	−0.395	< 0.001	−0.021 ± 0.007	−0.237	0.002	−0.026 ± 0.007	−0.261	< 0.001
Winter-Spring	−0.018 ± 0.137	0.009	0.896	−0.134 ± 0.199	−0.052	0.503	−0.092 ± 0.173	−0.037	0.598	0.160 ± 0.161	0.074	0.994	0.037 ± 0.180	0.015	0.836
25(OH)D (ng/mL)	0.011 ± 0.007	0.128	0.086	0.001 ± 0.010	0.011	0.893	0.017 ± 0.008	0.144	0.044	0.015 ± 0.008	0.148	0.052	0.015 ± 0.009	0.129	0.085
R^2^	0.138			0.033			0.205			0.099			0.127		

Abbreviation: B = unstandardized regression coefficients, β = standardized regression coefficients, DBP = diastolic blood pressure, eGFR = estimated glomerular filtration rate, FPG = fasting plasma glucose, HF = high frequency, LF = low frequency, LF/HF ratio = low frequency/high frequency ratio, SBP = systolic blood pressure, SE = standard error, TC = total cholesterol, VLF = very low frequency, WC = waist circumference, 25(OH)D = 25-hydroxyvitamin D. Age, sex, and seasons of test were included as covariate factors in a multivariate analysis. A *p*-value ˂ 0.00625 (0.05/8) was considered to indicate statistical significance because *p*-value was corrected by the Bonferroni correction for multiple comparisons.

**Table 4:** Association between vitamin D status and low heart rate variability.

25(OH)D Status	SDNN (<30 m/s)	RMSSD (<10 m/s)
	OR (95% Confidence Interval)	OR (95% Confidence Interval)
Model 1		
25(OH)D ≥ 15 ng/mL	1.00 (reference)	1.00 (reference)
25(OH)D < 15 ng/mL	2.38 (1.14–4.97)	1.60 (0.68–3.79)
Model 2		
25(OH)D ≥ 15 ng/mL	1.00 (reference)	1.00 (reference)
25(OH)D < 15 ng/mL	2.62 (1.19–5.72)	1.78 (0.72–4.39)
Model 3		
25(OH)D ≥ 15 ng/mL	1.00 (reference)	1.00 (reference)
25(OH)D < 15 ng/mL	3.07 (1.32–7.14)	1.86 (0.70–4.96)

Abbreviation: RMSSD = square root of the mean of sum of the square of differences between adjacent N-N intervals, SDNN = standard deviation of normal to normal, 25(OH)D = 25-hydroxyvitamin D. Model 1, unadjusted; Model 2, adjusted for gender and age; Model 3, adjusted for gender, age, seasons, alcohol use, current smoking status, regular exercise, waist circumference, body mass index, systolic blood pressure, diastolic blood pressure, total cholesterol, fasting plasma glucose and estimated glomerular filtration rate.

## 4. Discussion

The present study examined the relationship between serum vitamin D levels and HRV in healthy individuals. The SDNN was low in subjects with 25(OH)D deficiency, and 25(OH)D levels were associated positively with SDNN, and LF. This suggests that low 25(OH)D serum levels are associated with cardiac autonomic dysfunction, which may trigger a pathophysiological mechanism that increases CVD risk in healthy populations with vitamin D deficiency.

Vitamin D has direct effects on numerous cell types via actions on the vitamin D receptor (VDR) [1]. Although the heart is not considered a traditional target organ, growing evidence suggests that vitamin D plays crucial roles in heart structure and function. In animal studies, 1,25-dihydroxyvitamin D (1,25(OH)_2_D) impacted cardiac autonomic activity [19,20]. These studies demonstrated that 1,25(OH)_2_D deficiency resulted in accelerated rates of cardiac contraction and relaxation. In addition, VDR ablation led to cardiac fibrosis, hypertrophy and dysregulation of the renin-angiotensin system (RAS). The direct applicability of these findings in animals to humans is unclear, but VDR has been found in human cardiac tissue as a 55-kDa protein [21,22]. Patients with chronic kidney disease had a reduced capacity for converting 25(OH)D to 1,25(OH)_2_D due to decreased 1-α hydroxylase activity. These patients showed chronic RAS upregulation [23] and altered cardiac autonomic activity defined mainly by extreme vagal insufficiency [5]. Moreover, Adriana J *et al.* reported that 25(OH)D level was cross-sectionally related with higher B-type natriuretic peptide (BNP) in subjects with eGFR < 60 mL/min/1.73 m^2^, suggesting low 25(OH)D may be associated with growth and hypertrophy of cardiac cell, therefore may result in stimulated BNP secretion [24]. Studies of healthy populations have also shown that lower 25(OH)D levels were associated independently with a higher risk of SCD or CVD [3,15,25,26], suggesting that low vitamin D levels may also be an important and potentially treatable risk factor in populations without established pathologies. However, the molecular mechanisms responsible for the vitamin D deficiency associated with cardiac morbidity and mortality have not been fully elucidated. Our finding of a positive association between 25(OH)D levels and HRV suggests a possible mechanism for this phenomenon.

HRV depends on the sympathetic and parasympathetic effect on the sinus node and reflects changes in ANS activity and function. RMSSD and HF are the predominant responses to variations in parasympathetic tone. By contrast, SDNN and LF are influenced by both adrenergic and cholinergic activities and other physiological inputs. SDNN depends on a change in all HRV parameters and its decrease is associated with reduced function of the left ventricle [27]. TP level is similar to SDNN by affecting the control of the ANS and is generally decreased in individuals under chronic stress or with disease. Nolan *et al.* found prospectively that SDNN was an independently strong prognostic factor for CHF patients [28]. LF is an indicator of sympathetic activity regulation in the sinus node. Recent research has suggested that the LF component is reduced in patients with CHF; this decrease is related to a higher risk of sudden death, advanced disease, and progression to heart failure [29]. In our study, 25(OH)D levels were positively related with SDNN and LF, but not HF, indicating diminished sympathetic tone in subjects without pre-existing risk factors for CVD. The sympathetic nervous system has an important role in the regulation of energy homeostasis in humans [30]. Therefore, differences in sympathetic nervous system activity can cause variations in 24-h energy expenditure among individuals. Reduced activity of sympathetic tone associated with 25(OH)D deficiency may contribute to changes in cardiomyocyte energy expenditure. As mentioned previously, low vitamin D status may result in elevated RAS system activity, causing myocardial hypertrophy and arterial hypertension. In addition, vitamin D affects directly cardiomyocytes, including modulation of contractility, regulation of extracellular matrix turnover and anti-hypertrophic actions [31]. This may explain the higher risk of CVD in patients with vitamin D deficiency. By contrast, we found no evidence of a significant association between 25(OH)D levels and RMSSD and HF. Parasympathetic effects exert through rapidly dynamic control by acetylcholine influencing muscarinic receptors and are hereby reflected in the HF component of HRV. In cardiac disease, parasympathetic activation and its physiological effects decrease such as attenuation of vagal ganglionic transmission, change of muscarinic receptor composition and density, and reducing of acetylcholinesterase activity [32]. Because both RMSSD and HF component represent cardiac vagal nerve activity in the sinus node and electronic stability, a decrease in the parasympathetic nerve activity in the heart results in a decrease in RMSSD and HF component [16]. According to previous studies, decreased parasympathetic tone becomes a significant factor at more advanced stages of heart dysfunction [33,34,35]. Because we excluded patients with established risk factors for CVD, our findings suggest the 25(OH)D levels influence the early stage of pathophysiological changes in the heart. There are several evidences that vitamin D may be important in early process of atherosclerosis disease. Wang *et al.* and Giovannucci *et al.* reported an increased risk of CVD incidence among subjects with vitamin D deficiency in large prospective studies involving population without pre-existing CVD [15,26]. In contrast, prospective studies conducted in patients with stable coronary disease or advanced type 2 diabetes reported that baseline vitamin D levels did not predict cardiovascular events [36,37,38].

Limited studies of the link between vitamin D and HRV have been published. Only one study examined a relationship between vitamin D metabolites and modulation of the cardiac ANS in a healthy population [39]. Their findings of a significant association between low 25(OH)D levels and decreased baseline cardiac autonomic activity, low 1,25(OH)_2_D levels and unfavorable cardiosympathovagal changes during acute angiotensin II challenge are consistent with our results. Unfortunately, the findings could not be generalized to other studies due to the small sample size (*n* = 34). In addition, Metin Cetin *et al.* examined the relationship between vitamin D deficiency and autonomic imbalance in patients who had ischemic and non-ischemic dilated cardiomyopathy [40]. Surprisingly, they reported a stronger association between 25(OH)D levels and HRV, which reflects the activity of the ANS, in patients with non-ischemic rather than ischemic dilated cardiomyopathy. This finding suggests that vitamin D may play an important role in cardiomyocyte pathophysiology and that its deficiency may be more closely associated with the pathogenesis of non-ischemic rather than ischemic myocardial disease.

Our results also suggest that the positive association between 25(OH)D levels and ANS activity is involved in SCD pathogenesis. Interestingly, the association between vitamin D deficiency and risk for SCD was stronger in the population without than with CVD, as reported by Pilz *et al.* [41]. Altered myocardial calcium flux increased the risk of SCD related to vitamin D deficiency, suggesting a link to cardiac arrhythmia [42]. This hypothesis is supported by the positive association between 25(OH)D levels and corrected QT interval (QTc) in non-ischemic dilated cardiomyopathy patients [41]. Kim *et al.* also reported that calcitriol treatment decreases a prolonged QTc dispersion [42].

The present study had several limitations. First, our results do not represent the general population because we enrolled healthy individuals who visited a local university hospital. Secondly, determining the causal relationships between vitamin D and HRV parameters is difficult due to the cross-sectional nature of the study. Additionally, we were unable to assess the serum PTH level, which is an important determinant of vitamin D status. Hyperparathyroidism is linked to hypertrophy of cardiomyocytes and arterial stiffness and vitamin D deficiency may be predisposed to increased BP via elevated PTH and disturbed calcium homeostasis [43,44,45]. Moreover, the analysis of the VLF component could not be used to evaluate clinical implications because we examined HRV parameters only in the short-term (5 min). In such a short-term analysis, VLF does not provide adequate data as this band often reflects meaningless noise signals. HRV has been examined using electrocardiographic signals evaluated during short (2–5 min) and long (24-h) duration periods. We used a short-duration period because long-term electrocardiographic recording inhibits comparison of HRV parameters obtained during various activities such as exercise, sleep, and deep breathing. Finally, we could not apply the standardized forms of autonomic load, such as head-up tilt test, orthoclinostatic or orthostatic tests and deep breathing, in the examination of the HRV component.

Although the interest in vitamin D and its relationship to CVD risk has increased recently, evaluation of the risk of cardiac events in a healthy population with hypovitaminosis D but not established CVD risk factors, such as HTN, DM and dyslipidemia, is easily overlooked. Currently, many commercial devices that automate HRV measurements for research and clinical studies are available. These devices are simple and important tools for assessment of autonomic heart control and autonomic dysfunction.

## 5. Conclusions

In this cross-sectional study, vitamin D deficiency was associated independently with a risk of low HRV in a healthy population. This association remained after adjusting for age, gender, and season of 25(OH)D measurement. In addition, LF was lower in the 25(OH)D deficiency group than the non-deficient 25(OH)D level group. These observations suggest that sympathetic activities are attenuated in vitamin D deficiency. Although the study included a small population at a single center, it increases our understanding of the etiology and pathophysiology of the heart in patients with hypovitaminosis D. Therefore, 25(OH)D levels may contribute to autonomic dysfunction by a pathophysiological mechanism that may increase the risk of cardiac adverse events in healthy populations with vitamin D deficiency. Maintaining a sufficient 25(OH)D level may reduce the risk of CVD through favorable changes in cardiac autonomic function in populations with hypovitaminosis D. Further experimental studies are needed to identify the effect of vitamin D supplementation on HRV in healthy populations.
